# Regenerative Endodontic Therapy of an Immature Permanent Molar With Necrotic Pulp and Symptomatic Apical Periodontitis Using Platelet‐Rich Fibrin: A 52‐Month Follow‐Up Case Report

**DOI:** 10.1155/crid/2692780

**Published:** 2026-08-26

**Authors:** Bikash Chaurasia, Tahsin Raquib Abonti, Mark Low Jun Fay, Wan Badrina Binti Wan Bunyamin, Goh Michelle

**Affiliations:** ^1^ Department of Pediatric Dentistry, School of Dentistry, Lincoln University College, Petaling Jaya, Selangor, Malaysia, lincoln.edu.my; ^2^ Department of Orthodontics and Dentofacial Orthopedics, Pioneer Dental College and Hospital, Dhaka, Baridhara, Bangladesh; ^3^ Department of Endodontics, School of Dentistry, Lincoln University College, Selangor, Malaysia, lincoln.edu.my; ^4^ Undergraduate student, School of Dentistry, Lincoln University College, Petaling Jaya, Selangor, Malaysia, lincoln.edu.my

**Keywords:** apical periodontitis, case report, immature permanent molar, necrotic tooth, platelet-rich fibrin, regenerative endodontic therapy, revascularization

## Abstract

**Background:**

Regenerative endodontic therapy (RET) is a biologically based, conservative treatment approach for immature permanent teeth with necrotic pulp that aims to induce physiologic root closure and thickening of root dentin. This case report describes the 52‐months clinical and radiographic outcome of RET using platelet‐rich fibrin (PRF) as a scaffold in an immature permanent molar with necrotic pulp and apical periodontitis.

**Case Report:**

A healthy 7‐year‐old boy presented with pain and a decayed tooth in the lower left back region of the jaw of 3–4 months′ duration. Swelling in the same region 15 days earlier had resolved spontaneously. Clinical examination revealed a deep carious lesion in tooth 36. Oral hygiene was poor, and the tooth failed to respond to cold and electric pulp testing (EPT), with mild tenderness to percussion and palpation. Radiographic examination showed open apices, thin dentinal walls, and a periapical radiolucency associated with the mesial root. After informed consent, local anesthesia was administered, and under rubber dam isolation, caries was removed and an access was cavity prepared. The canals were located, irrigated with sodium hypochlorite, and dressed with triple antibiotic paste for 4 weeks. At the second visit, intracanal bleeding could not be induced from the periapical region despite repeated attempts; therefore, approximately 5 mL of venous blood was collected from the left antecubital vein to prepare PRF, which was placed into the canals to approximately 1 mm beyond the apices using a hand plugger, with the coronal extent to the level of the cementoenamel junction. A 3‐mm layer of white mineral trioxide aggregate (MTA) was placed over the PRF, and the tooth was restored the following day with glass ionomer cement and composite resin. Clinical and radiographic follow‐ups were conducted at 9, 18, 24, 36, and 52 months. Progressive healing was observed, including resolution of the periapical lesion, increased root length, thickening of the root dentin, and continued apical closure. The tooth responded positively to cold testing at 18 months and to EPT at 24 months.

**Conclusion:**

In this case, RET using PRF as a scaffold was associated with favorable long‐term clinical and radiographic outcomes, including resolution of periapical pathology, progressive apical closure, and thickening of the root dentinal walls, in an immature permanent molar with necrotic pulp and apical periodontitis. These findings suggest PRF may be a useful scaffold option for RET in immature molars; however, as a single case, this report cannot establish the efficacy or superiority of PRF over conventional blood clot or platelet‐rich plasma (PRP) scaffolds, and prospective controlled studies are needed.

## 1. Introduction

A vital, healthy dental pulp is essential for continued root development and maturation of an immature permanent tooth [1]. Pulp necrosis in these teeth may follow dental trauma, most often in anterior teeth, or a long‐standing deep carious lesion, more often in molars, and it is a recognized consequence of molar incisor hypomineralization (MIH) [2]. When necrosis occurs before root development is complete, the result is an immature tooth with an open apex and thin, fracture‐prone dentinal walls [3, 4], which presents a considerable challenge for canal disinfection and for inducing an intracanal blood clot [5]. Treating such teeth is technique‐sensitive: pediatric dentists and endodontists must balance biological objectives, including pulp survival and continued root maturation, against practical ones, including a young child’s cooperation, anxiety, and ability to attend repeated follow‐up visits.

Traditionally, apexification with calcium hydroxide was the standard treatment for immature teeth with open apices and thin dentinal walls, using long‐term intracanal medication to promote formation of an apical hard‐tissue barrier [6]. However, prolonged intracanal calcium hydroxide has been associated with an increased risk of root fracture [6]. Torabinejad and Chivian subsequently introduced single‐visit apexification using mineral trioxide aggregate (MTA), which reliably creates an artificial apical barrier and supports resolution of periapical disease [7]. Neither approach, however, promotes further thickening or lengthening of the root, so the tooth remains structurally weaker than a tooth with a completed root [6, 7].

Regenerative endodontic therapy (RET) was developed to address this limitation by aiming to restore a pulp–dentin complex capable of continued root maturation rather than simply sealing the apex. The biological rationale traces back to Nygaard‐Østby′s early histological work on the role of the blood clot in endodontic healing [8]. Iwaya et al. subsequently described a clinical protocol representing one of the earliest reported applications of this approach in a tooth with apical periodontitis and a sinus tract [9], and in 2004 Banchs and Trope popularized the term “revascularization” and proposed a standardized clinical protocol [10]. We note this only as a clarification of terminology, since the underlying biological concept predates both reports [8]. Case reports and case series have generally described favorable radiographic outcomes with RET, including increased root length and wall thickness and apical closure [9, 11, 12], although unfavorable outcomes, including failed revascularization and histologically confirmed failure, have also been reported [13, 14].

An intracanal blood clot is central to conventional RET; it acts as a scaffold, recruits stem cells from the periapical region, and provides growth factors that support continued root development [15]. A blood clot alone, however, has recognized limitations as a scaffold, prompting exploration of alternative materials [16]. Platelet‐rich plasma (PRP) and platelet‐rich fibrin (PRF) are autologous platelet concentrates that have been investigated as alternatives. PRP provides structural support together with concentrated platelets and growth factors [17], whereas PRF, a second‐generation platelet concentrate first described by Choukroun and colleagues [18, 19] forms a dense fibrin matrix that releases growth factors more gradually than PRP, without requiring anticoagulants or exogenous activators [20]. In a 2018 meta‐analysis, Murray reported that PRP and PRF were each associated with a higher rate of apical closure at 1 year than conventional blood‐clot revascularization [21], and more recent systematic reviews and meta‐analyses of randomized trials have continued to compare platelet concentrates with blood‐clot scaffolds, with some but not all outcome measures favoring platelet concentrates [22].

RET is now well established for immature anterior teeth [23, 24]. Its application in immature molars has been reported less frequently, in a small number of case reports and case series [11, 25–27], and a standardized, molar‐specific treatment protocol has not been established. Current clinical guidance, including that of the American Association of Endodontists (AAE), was developed primarily for anterior teeth and is commonly extrapolated to molar cases [28]. PRF specifically has been used as a scaffold in immature molars in a six‐tooth case series reporting periapical healing and root maturation [26], and in an individual case followed for 48 months, the longest previously reported follow‐up for PRF‐based RET in a molar, in which early apical closure was later complicated by extensive intracanal calcification and recurrent caries necessitating retreatment [27]. Comparable long‐term outcome data for PRF‐based RET in molars therefore remain limited to a small number of reports, and, to our knowledge, no previously published case has documented a favorable outcome without need for retreatment at a follow‐up interval as long as 52 months.

This case report was prepared in accordance with the Preferred Reporting Items for Case Reports in Endodontics (PRICE) 2020 guideline [29] and the CARE (CAse REport) 2013 guideline [30], and describes the 52‐month clinical and radiographic outcome of RET using PRF as a scaffold in an immature mandibular first molar with necrotic pulp and symptomatic apical periodontitis. It additionally documents an intraoperative, reactive switch from a planned blood‐clot to a PRF scaffold after failed intracanal bleeding, a clinical scenario that arises in practice but is seldom described in detail, together with calibrated, examiner‐assessed quantitative radiographic measurements at each follow‐up interval. The findings are discussed in the context of the wider RET, molar, and PRF literature, together with the limitations inherent to a single case.

## 2. Report

A healthy 7‐year‐old boy reported to the Department of Pediatric Dentistry with a chief complaint of pain and a decayed tooth in the lower left back region of 3–4 months′ duration. The pain was mild to moderate, dull, intermittent, aggravated by food lodgement, and relieved with ibuprofen, as reported by his father. He also had a history of swelling in the same region 15 days earlier, which resolved spontaneously without fever or pus discharge. No previous dental treatment had been sought, and the patient had been taking over‐the‐counter analgesics regularly. His medical history was noncontributory.

**Figure 1:**
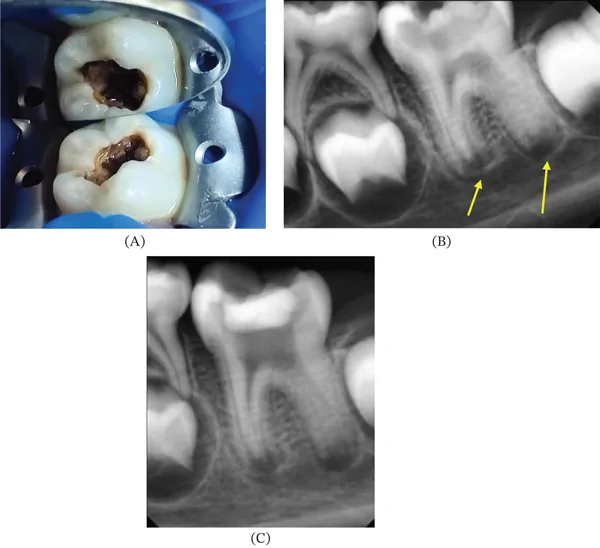
(A) Preoperative intraoral clinical photograph showing tooth 36 (deeply carious) under rubber dam isolation. (B) Diagnostic intraoral periapical (IOPA) radiograph showing tooth 36 (open apices visible). (C) IOPA radiograph after triple antibiotic paste placement into the canals and coronal seal with Cavit.

Intraoral examination revealed a deep carious lesion in tooth 36 (Figure 1A). Cold testing and EPT elicited no response; mild tenderness to percussion and palpation was noted. Periodontal probing depths were within normal limits. Radiographic examination showed an immature root with thin walls and an open apex in both roots (Cvek stage 4—wide open apical foramen with nearly complete root length) [4], a large coronal radiolucency involving enamel, dentin, and pulp, and a periapical radiolucency measuring approximately 2 × 4 mm^2^ around the mesial root (Figure 1B). This measurement was an approximate visual estimate on a two‐dimensional periapical radiograph, without a calibrated reference or correction for magnification or geometric distortion, and may in part reflect periodontal ligament space widening rather than a discrete, well‐defined lesion.

A diagnosis of pulp necrosis with symptomatic apical periodontitis was made on the combination of negative sensibility responses, clinical signs, and radiographic findings. False‐negative responses to cold testing and EPT are recognized in immature teeth with open apices owing to incomplete neural development, so the possibility of partial residual pulp vitality could not be entirely excluded on sensibility testing alone; the diagnosis in this case therefore relied on the overall clinical and radiographic picture rather than sensibility testing in isolation. RET with an induced blood clot scaffold from the periapical region was planned, and the procedure was explained in detail to the parent; written informed consent was obtained, including for the possibility of an alternative scaffold if blood clot induction failed. The possibility of PRF and venipuncture as a contingency, and the need for child assent before venipuncture, were also discussed at this initial consent stage, prior to the start of treatment.

### 2.1. First Visit

Local anesthesia (2% lidocaine with 1:100,000 epinephrine) was administered, followed by rubber dam application. Carious dentin and unsupported enamel were removed with a round bur and air rotor. After access cavity preparation, the mesiobuccal (MB), mesiolingual (ML), and distal (D) canals were located. Minimal instrumentation was performed, and each canal was irrigated with 20 mL of 1.5% sodium hypochlorite (NaOCl) for 5 min using a 30‐gauge side‐vented irrigation needle placed 1–2 mm short of the working length. This NaOCl concentration was selected to balance adequate antimicrobial action against the risk of cytotoxicity to surviving apical papilla stem cells [31]. Working lengths were determined with a #15 K‐file for the mesial canals (MB = 18 mm, ML = 18 mm) and a #20 K‐file for the D canal (D = 19 mm) using tactile and radiographic methods. The ML canal file initially appeared overextended radiographically and was adjusted using the radiographic apex of the MB canal as a reference. The canals were dried with paper points, and a triple antibiotic paste (TAP) of ciprofloxacin, metronidazole, and minocycline in equal proportions with normal saline, at a final concentration of 1 mg/mL, was placed to 2 mm short of the apex using a lentulo spiral. This low concentration was deliberately selected because higher concentrations of TAP (>1 mg/mL) are cytotoxic to stem cells of the apical papilla (SCAP) in vitro [32], consistent with AAE guidance cautioning against high‐concentration TAP [28]. To reduce the risk of crown discoloration from minocycline, the paste was kept 1 mm below the cementoenamel junction (CEJ) and sealed temporarily with Cavit. A postoperative radiograph was obtained (Figure 1C), and the patient was recalled after 4 weeks.

### 2.2. Second Visit

The patient was asymptomatic. Local anesthesia with 2% lidocaine without epinephrine was given, a vasoconstrictor‐free anesthetic was deliberately chosen at this visit to avoid inhibiting intracanal bleeding during attempted blood clot induction followed by rubber dam isolation. The access cavity was reopened, and each canal was irrigated with 20 mL of 17% ethylenediaminetetraacetic acid (EDTA) for 5 min, followed by saline to flush residual antibiotic paste. In addition to smear layer removal, 17% EDTA releases dentin‐matrix‐bound growth factors, including TGF‐*β*1, and has been shown to support SCAP survival better than NaOCl alone, favoring a more regeneration‐conducive canal environment [31, 33]. The canals were dried with paper points, and intracanal bleeding was attempted by over‐instrumenting 2 mm beyond the apex with a #20 K‐file. Despite multiple attempts, no bleeding could be induced in any canal.

The reason for this failure was not established with certainty. Possible contributing factors include apical fibrosis or calcification of the periapical tissue, insufficient instrumentation beyond the apical foramen, or a genuinely limited volume of vascularized periapical tissue despite radiographic evidence of an open apex. Because assessment and instrumentation of a blood clot in a molar is also technically more difficult than in an anterior tooth owing to limited visibility and access, PRF was selected as an alternative scaffold. The change in plan was explained to the parents, and separate written informed consent for venous blood collection was obtained intraoperatively.

**Figure 2:**
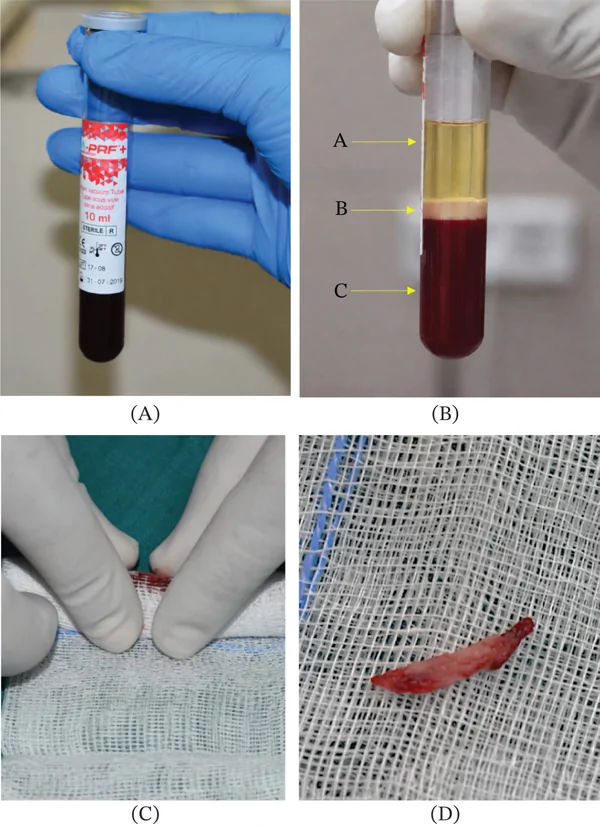
(A) 5 mL of whole blood collected in a glass vacutainer. (B) After centrifugation: A—platelet‐poor plasma, B—PRF, C—red blood cells. (C) Excess fluid being expressed from the PRF clot. (D) Final PRF membrane on sterile dry gauze.

Approximately 5 mL of whole blood was collected by a trained phlebotomist from the left antecubital vein using an 18‐gauge needle and transferred into a sterile glass vacutainer without anticoagulant (Figure 2A). Appropriate assent was obtained from the child, in addition to parental consent, before the procedure, and the child remained under local anesthesia and rubber dam isolation throughout venous blood collection and PRF preparation. The interval between venipuncture and the start of centrifugation was under 2 min, consistent with the requirement for immediate centrifugation to preserve PRF quality. The tube was immediately centrifuged (Benchmark centrifuge, Raptor Supplies SG) at 3500 rpm for 15 min using a rotor with an effective radius of 73 mm, corresponding to a relative centrifugal force of approximately 1006×g, producing three layers (Figure 2B): platelet‐poor plasma (PPP) superiorly, a PRF clot centrally, and red blood cells inferiorly. The PPP was discarded with a pasteur pipette, and the PRF clot was retrieved with a sterile tweezer and compressed with sterile dry gauze to obtain a PRF membrane (Figure 2C).

**Figure 3:**
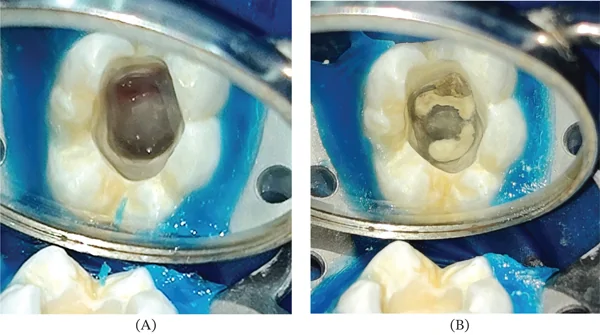
(A) PRF membrane placed in all three canals to the level of the CEJ. (B) MTA placed over the PRF membrane in all three canals.

The PRF membrane was rolled to the established working length (Figure 2D) and visually divided into three approximately equal‐length segments with a sterile scalpel blade, one for each canal (MB, ML, D); the length and estimated volume of each segment were checked against the corresponding canal′s working length and judged adequate as a scaffold. Given the freehand, technique‐sensitive nature of this division, exact equivalence of PRF volume between canals cannot be guaranteed. Managing the sticky consistency of the PRF membrane, it was packed to approximately 1 mm beyond the working length using a hand plugger dipped frequently in sterile distilled water, and coronally to the level of the CEJ (Figure 3A). The extent of extrusion was judged clinically using tactile sensation and the working‐length reference rather than confirmed radiographically or under magnification. A 3‐mm layer of white MTA was placed directly over the PRF scaffold (Figure 3B), followed by a moist cotton pellet, and the tooth was temporarily restored with Cavit.

**Figure 4:**
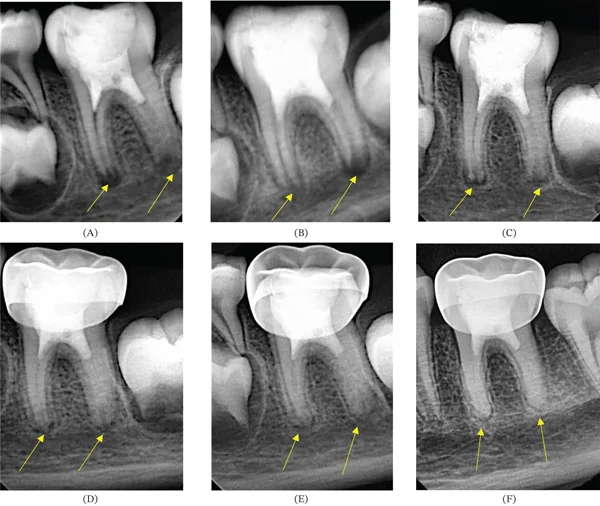
(A) After final restoration with glass ionomer cement and composite resin. (B) Nine‐month follow‐up: resolution of periapical radiolucency, increasing root length, and thickening root width. (C) Eighteen‐month follow‐up: increasing root length and thickening root width, with apical narrowing. (D) Twenty‐four‐month follow‐up: partial obliteration of canal space in both roots. (E) Thirty‐six‐month follow‐up: near‐complete obliteration of canal space and apical root closure. (F) Fifty‐two‐month follow‐up: complete obliteration of canal space and apical root closure.

The patient returned the following day to confirm MTA setting, and the access cavity was permanently restored using a sandwich technique with glass ionomer cement and composite resin (Figure 4A).

### 2.3. Follow‐Up

Clinical and radiographic follow‐up was performed at 9, 18, 24, 36, and 52 months (Figures 4B–4F). The first scheduled review took place at 9 months; earlier reviews at 1, 3, and 6 months, recommended by several RET protocols to detect early failure, reinfection, or adverse events, were not performed in this case, as the patient and parents were unable to attend during that period owing to family commitments and had some urgency to return to their hometown although informal interim contact with the family was maintained despite no radiograph being taken. A stainless steel crown (SSC) was placed at the 24‐month review; the parents had initially been reluctant to proceed with a full‐coverage restoration and wished to seek a second opinion from other family members regarding crown treatment, which resulted in a substantial delay before the SSC was eventually placed.

Positive responses to cold testing were recorded at 18 months and to EPT at 24 months, reported as comparable to the contralateral first molar. The EPT reading was 42 units for the study tooth and 28 units for the contralateral control tooth, recorded with the electrode placed at the MB cusp tip of both teeth under rubber dam isolation. EPT was performed immediately before SSC placement at this same 24‐month visit, so this particular reading is not confounded by the crown; EPT through a full‐coverage metal restoration is nonetheless recognized to be unreliable owing to dispersion of current through the metal [34], which is relevant to interpreting any sensibility testing of this tooth at later visits. Both cold and EPT responses indicate sensibility, an intact neural response rather than confirmed histological pulp vitality or a regenerated, functional pulp–dentin complex, which can only be established histologically. Responses to percussion and palpation remained within normal limits throughout follow‐up, and periodontal probing depths remained normal.

Radiographic evaluation of root length, root wall thickness, and apical diameter was performed independently by two examiners (a pediatric dentist and an endodontist) using ImageJ software with a calibrated reference, at baseline and at each follow‐up interval; the measured values and percentage change from baseline are summarized in (Table 1). Disagreements were resolved by discussion and consensus; formal interexaminer reliability (e.g., intraclass correlation coefficient) was not calculated. Radiographic standardization across the 52‐month follow‐up period was achieved using a paralleling technique with a customized positioning jig, since angulation can otherwise materially affect apparent root length and wall‐thickness measurements on serial radiographs. Follow‐up radiographs showed resolution of the periapical lesion, continued thickening of the root dentinal walls, progressive apical closure with narrowing of the apical foramen, and progressive obliteration of the canal space from the 24‐month review onward.

**Table 1:** Summary of clinical and radiographic follow‐up findings (radiographic measurements by ImageJ with calibrated reference).

Follow‐up	Root length MB/ML/D (mm)	^a^Wall thickness overall (mm)	^b^Apical diameter overall (mm)	% change from baseline (a/b)	Cold test	EPT	Clinical findings
Baseline	18/18/19	0.80	1.70	—	Negative	Negative	Deep caries, tenderness to percussion/palpation
9 months	18.1/18.2/18.1	0.90	1.40	12.5 / −17.6	Negative	Negative	Resolution of periapical radiolucency; asymptomatic
18 months	18.2/18.2/18.2	1.00	1.00	25 / −41.1	Positive	Negative	Continued apical narrowing; asymptomatic
24 months	18.6/18.5/18.6	1.10	0.70	37.5 / −58.8	Positive	Positive^c^	SSC placed; partial canal obliteration; asymptomatic
36 months	18.8/18.9/18.8	1.20	0.50	50 / −70.5	NP	NP	Near‐complete canal obliteration; asymptomatic
52 months	19.4/19.6/19.6	1.50	0.20	87.5 / −88.2	NP	NP	Complete canal obliteration; asymptomatic

*Note:* (a/b)‐ % change in wall thickness and apical diameter each.

Abbreviation: NP, not performed.

^a^Wall thickness.

^b^Apical diameter.

^c^EPT performed before stainless steel crown placement.

## 3. Discussion

Treating immature permanent teeth in very young patients is both technique‐sensitive and challenging for pediatric dentists. Although the general principles of RET, including canal disinfection and intracanal medication, are broadly similar across indications, outcomes can differ between traumatized and carious teeth, possibly reflecting differing degrees of damage to the periodontal ligament and Hertwig′s epithelial root sheath (HERS) following trauma [10, 35].

Complete disinfection of the root canal system is critical for successful RET [25]. TAP, comprising ciprofloxacin, metronidazole, and minocycline, is effective against the complex microbial flora present in the root canal system and deeper dentin [11, 36], but its major disadvantage is crown discoloration from minocycline [36]. In this case, TAP was kept 1 mm below the CEJ to limit discoloration, which was judged a lesser concern in a molar than an anterior tooth. Because TAP cytotoxicity to SCAP is concentration‐dependent [32], current AAE guidance favors a low‐concentration formulation, a double antibiotic paste omitting minocycline, or calcium hydroxide as alternatives where discoloration or stem cell toxicity is a greater concern [28]. Consistent with this guidance, a low‐concentration (1 mg/mL) TAP formulation was deliberately used in this case rather than a conventional high‐concentration paste, and we would continue to favor this low‐concentration approach or a double antibiotic paste omitting minocycline where discoloration risk is a greater concern in similar future cases.

The concentration of irrigant used may reasonably be adjusted according to the extent of periapical pathology. In this case, 1.5% NaOCl was used at the first visit, sufficient to maintain a sterile environment and dissolve necrotic debris while limiting cytotoxicity to viable apical stem cells [31]; more extensive or long‐standing periapical lesions may warrant a higher NaOCl concentration, balanced against the risk of damaging viable apical tissue.

Treatment in this case broadly followed AAE clinical recommendations for regenerative procedures [28]. It should be emphasized, however, that current high‐level evidence has not established the superiority of RET over MTA apexification, particularly in immature molars. RET has a plausible biological advantage in promoting continued root maturation rather than only achieving an apical barrier, and retrospective cohort data show RET more consistently associated with continued root development than apexification, with broadly comparable rates of clinical and radiographic success between the two approaches [24]. Earlier claims in the literature and in the original submission of this report describing RET as ensuring “better and more predictable outcomes” than apexification should therefore be read as describing a plausible biological advantage rather than an established clinical superiority.

### 3.1. Failure of Intracanal Bleeding

Failure to induce intracanal bleeding, as occurred at the second visit in this case, has been described previously, particularly in molars, where visibility and access are more limited than in anterior teeth [11, 25]. Proposed explanations include apical fibrosis or calcification of periapical tissue, inadequate instrumentation beyond the apical foramen, and a genuinely limited volume of vascularized periapical tissue despite radiographic evidence of an open apex. Because the shift to PRF in this case was a reactive, intraoperative decision rather than part of a pre‐planned protocol, it raises a broader question for future practice; whether PRF should be prepared prophylactically before attempting blood clot induction in molars, given the additional burden an unplanned venipuncture places on a young child and family, and whether informed consent and, where developmentally appropriate, child assent for venipuncture should be obtained routinely before any RET procedure in molars rather than only if bleeding fails. We raise this as a candidate protocol modification for future study rather than as an established recommendation, and note that the comparative failure rate of blood clot induction in molars versus anterior teeth is not well quantified in the current literature.

### 3.2. Root Development Stage and Prognosis

The tooth in this case was classified as Cvek Stage 4, indicating nearly complete root length with only the apical foramen remaining open [4]. Root development stage influences RET outcomes, with teeth at earlier stages (Cvek 1–3) generally showing greater capacity for further root lengthening than teeth at Stage 4 [4, 24]. The potential for further root elongation in this case was therefore inherently limited from the outset, and the favorable radiographic changes observed most likely reflect increased dentinal wall thickness and progressive apical closure rather than substantial further increase in root length. This should be considered when interpreting the outcome of this case and when comparing it with cases at earlier stages of root development.

### 3.3. PRF Biology and Handling

PRF differs from PRP in forming a dense, trimolecular fibrin matrix that entraps leukocytes and platelets, and its preparation does not require anticoagulants or exogenous activators [18, 19]. In vitro work has shown that PRF releases key growth factors including transforming growth factor‐*β*1, platelet‐derived growth factor‐AB, and vascular endothelial growth factor gradually over the period following preparation, in contrast to the more rapid release seen with PRP [20]. Leukocytes retained within PRF are also thought to contribute anti‐inflammatory activity and support angiogenesis [18, 19]. Handling PRF is technique‐sensitive; its jelly‐like consistency and tendency to adhere to instruments make precise, reproducible placement within a narrow canal space more difficult than with a liquid blood clot, and outcomes may depend in part on operator experience [37].

### 3.4. Intentional Apical Extrusion of PRF

PRF was intentionally packed approximately 1 mm beyond the working length in this case. We are not aware of controlled evidence specifically supporting intentional extrusion of scaffold material beyond the apical foramen in RET, and this aspect of the protocol should be regarded as an individualized clinical judgment rather than an established, evidence‐based technique. Potential risks of periapical extrusion include a foreign‐body‐type inflammatory response and delayed resorption; proponents might argue that direct scaffold contact with periapical tissue could support cellular ingrowth into the canal space, particularly where bleeding could not be induced. Given the absence of supporting evidence, we would not recommend intentional apical extrusion of PRF as routine practice on the basis of this case, and highlight it as an area requiring further study rather than a validated element of the protocol.

### 3.5. MTA Placed Directly Over PRF

White MTA was placed directly over the PRF scaffold. MTA has a high pH during initial setting, and it is biologically plausible that direct contact with an alkaline‐setting material could affect growth factors or cellular elements in the immediately adjacent PRF membrane. We are not aware of published data directly quantifying this interaction in a clinical RET context. This potential interaction did not appear to compromise the radiographic outcome in this case, but it is acknowledged as an unresolved question rather than a validated aspect of the protocol.

### 3.6. Canal Obliteration

Progressive canal‐space obliteration was observed from the 24‐month review onward. Although sometimes reported as a favorable sign in the RET literature, canal obliteration is increasingly recognized as a controversial finding; it does not necessarily represent regeneration of a functional pulp–dentin complex, may instead reflect reparative or dystrophic hard‐tissue deposition, and can complicate future retreatment should the tooth later require root canal therapy [38]. Therefore, this case should be interpreted as demonstrating a radiographically favorable but biologically ambiguous outcome rather than confirmed pulp–dentin regeneration.

### 3.7. Nature of the Regenerated Tissue

Sensibility appeared to be present in the treated tooth from 18 months onward; however, positive responses to cold and electric testing indicate a functioning sensory response rather than confirmed histological pulp vitality or true regeneration of an odontoblast‐mediated pulp–dentin complex [39]. Animal and human histological studies of RET have shown that tissue occupying the canal space after revascularization more closely resembles cementum‐ or periodontal‐ligament‐like tissue, or reparative hard tissue potentially mediated by surviving HERS, rather than a fully regenerated, functional pulp [39–41]. Without histological confirmation, which was not obtainable in this clinical case, the precise nature of the tissue responsible for the observed continued root maturation and apical closure cannot be established, and we have therefore avoided describing the outcome as confirmed regeneration of vital pulp tissue.

## 4. Limitations

This report has several limitations inherent to a single case. First, conclusions about the relative efficacy or superiority of PRF compared with conventional blood clot or PRP scaffolds cannot be drawn from one case, and causality between the PRF scaffold and the observed outcome cannot be established. Second, no histological confirmation of the tissue occupying the canal space was obtained; sensibility testing indicates a functional neural response but not confirmed pulp regeneration. Third, radiographic assessment was limited to two‐dimensional periapical radiographs; no cone beam computed tomography (CBCT) was performed, which would have allowed more accurate three‐dimensional assessment of root length, wall thickness, apical diameter, and periapical healing, and would have avoided the geometric distortion and magnification inherent to periapical radiography [42]. Fourth, although quantitative radiographic measurements are now reported (Table 1), formal interexaminer reliability statistics (e.g., an intraclass correlation coefficient) were not calculated. Fifth, early follow‐up at 1, 3, and 6 months was not performed, so the early healing trajectory and any early complications cannot be described. Sixth, EPT performed after stainless steel crown placement is of uncertain reliability owing to current dispersion through the metal restoration [34], which is relevant to any future sensibility testing of this tooth even though the 24‐month reading reported here preceded crown placement. Seventh, the intraoperative shift from blood clot to PRF was a reactive decision rather than part of a preplanned protocol, which limits the reproducibility of this specific treatment sequence. Finally, long‐term functional follow‐up including monitoring through adolescence as masticatory load increases, and eventual replacement of the stainless steel crown with a definitive restoration remains desirable; however, the family has indicated that further review visits may not be feasible because of the financial and logistical burden of travel, which itself illustrates a practical barrier to long‐term follow‐up in RET research that is rarely discussed in the literature.

## 5. Ethical Considerations

Written informed consent for treatment, including the possible use of PRF and venous blood collection, was obtained from the patient′s parent. In addition, developmentally appropriate assent was sought from the child before venipuncture, and the child remained under local anesthesia and rubber dam isolation while consent was being obtained intraoperatively and blood was drawn, consistent with ethical requirements for invasive procedures in pediatric patients.

## 6. Conclusion

In this case, RET using PRF as a scaffold was associated with a favorable clinical and radiographic outcome over 52 months of follow‐up in an immature mandibular molar with necrotic pulp and symptomatic apical periodontitis, including resolution of the periapical lesion, thickening of the root dentinal walls, and progressive apical closure. Root development stage (Cvek Stage 4) at the time of treatment inherently limited the potential for further root lengthening, and canal‐space obliteration observed on follow‐up radiographs should be interpreted as a radiographically favorable but biologically ambiguous finding rather than confirmed pulp–dentin regeneration. As a single case, this report cannot establish the efficacy or superiority of PRF over conventional blood clot or PRP‐based scaffolds in RET, particularly in molars; the intraoperative change from a planned blood clot to PRF after failed bleeding induction further illustrates that RET in molars remains technique‐sensitive and does not always proceed as planned. We suggest that this 52‐month follow‐up adds useful long‐term data to a literature in which molar RET outcomes beyond 24 months remain scarce, and that prospective, adequately powered studies ideally comparing PRF with conventional blood clot and PRP scaffolds using standardized radiographic (including CBCT where feasible) and, where possible, histological outcome measures are needed before firm conclusions about the optimal scaffold for RET in immature molars can be drawn.

## Author Contributions

**Bikash Chaurasia:** conceptualization of the case report, clinical management of the patient, case record, interpretation of clinical and radiographic findings, recording follow‐up data, manuscript drafting and literature review, critical revision of the manuscript, final draft of the manuscript. **Tahsin Raquib Abonti:** clinical case discussion, interpretation of radiographic findings, radiographic image analysis, manuscript drafting, critical revision of the manuscript, final draft of the manuscript. **Mark Low Jun Fay:** clinical case discussion, clinical management of the patient, case record, interpretation of clinical and radiographic findings, recording follow‐up data, revision of the manuscript. **Wan Badrina Binti Wan Bunyamin:** assistance with patient management, acquisition of clinical data and images, recording follow‐up data, manuscript editing, and review of the final manuscript. **Goh Michelle:** assistance with patient management, acquisition of clinical data and images, recording follow‐up data, manuscript editing, and review of the final manuscript.

## Funding

No funding was received for this manuscript.

## Consent

Written informed consent was obtained from the patient′s parent for publication of this case report and any accompanying clinical images and radiographs.

## Conflicts of Interest

The authors declare no conflicts of interest.

## Patient Perspective

The patient′s father reported that the child experienced complete resolution of pain and was able to chew comfortably following treatment. He expressed satisfaction that the tooth could be preserved without extraction and appreciated the minimally invasive approach. During follow‐up visits, the child remained asymptomatic and maintained normal oral function. The parent was pleased with the treatment outcome and the continued development of the immature tooth.

## Supporting information


**Supporting Information** Additional supporting information can be found online in the Supporting Information section. This case report was prepared in accordance with the Preferred Reporting Items for Case Reports in Endodontics (PRICE) 2020 guideline and the CARE (CAse REport) 2013 guideline.

## Data Availability

The data that support the findings of this study are available on request from the corresponding author. The data are not publicly available due to privacy or ethical restrictions.
