# Supplementary material for: Regenerative Endodontic Therapy of an Immature Permanent Molar With Necrotic Pulp and Symptomatic Apical Periodontitis Using Platelet‐Rich Fibrin: A 52‐Month Follow‐Up Case Report
Source: Case Rep Dent. 2026 Aug 26;2026:2692780. doi: 10.1155/crid/2692780 (PMC13509411; doi:10.1155/crid/2692780)
Supplement: Supplementary file 1 [file CRID-2026-2692780-s001.zip › 2692780/CARE_2013_Checklist_FINAL.docx]

**CARE 2013 Checklist**

Manuscript: “Regenerative Endodontic Therapy of an Immature Permanent Molar with Necrotic Pulp and Symptomatic Apical Periodontitis Using Platelet-Rich Fibrin: A 52-Month Follow-Up Case Report.”

Journal: Case Reports in Dentistry.

Gagnier JJ, Kienle G, Altman DG, Moher D, Sox H, Riley D; CARE Group. The CARE guidelines: consensus-based clinical case reporting guideline development. Headache. 2013;53(10):1541–1547.

| **Item** | **Description** | **Reported?** | **Location (document, page)** |
| --- | --- | --- | --- |
| 1 | Title — the diagnosis or intervention of primary focus followed by the words “case report”. | **Reported** | Manuscript, Title page, p.1 |
| 2 | Key words — 2 to 5 key words that identify diagnoses or interventions in this case report (including “case report”). | **Reported** | Manuscript, Keywords, p.3 |
| 3 | Abstract (structured or unstructured): introduction/novelty; patient's main concerns and clinical findings; primary diagnoses, interventions and outcomes; conclusion/take-away lessons. | **Partially reported** | Manuscript, Abstract, p.2–3 (findings, diagnosis, intervention, outcome and conclusion all present; explicit novelty/gap statement is in the Introduction, p.5, rather than the Abstract itself) |
| 4 | Introduction — briefly summarizes why this case is unique, with medical literature references. | **Reported** | Manuscript, Introduction, p.4–6 |
| 5 | Patient Information — de-identified specific information; main concerns/symptoms; medical, family and psychosocial history including relevant genetic information; relevant past interventions and outcomes. | **Partially reported** | Manuscript, Report, p.6 (age, gender, chief complaint, medical/dental history reported; family, psychosocial and genetic history not mentioned — likely not relevant to this case) |
| 6 | Clinical Findings — significant physical examination and important clinical findings. | **Reported** | Manuscript, Report, p.6 (intraoral findings, cold/EPT testing, percussion/palpation, probing depths) |
| 7 | Timeline — historical and current information from this episode of care, organized as a timeline (figure or table). | **Reported** | Table 1 document, p.1 (referenced in Manuscript, Follow-Up, p.10, and Limitations, p.14; Table 1 file itself not reviewed here — see note); narrative timeline throughout Manuscript Report/Follow-Up, p.6–10 |
| 8 | Diagnostic Assessment — diagnostic methods; diagnostic challenges; diagnosis (including other diagnoses considered); prognostic characteristics. | **Partially reported** | Manuscript, Report, p.6–7 (methods, diagnosis, and false-negative-sensibility challenge reported; alternative differential diagnoses not explicitly enumerated); Discussion, p.12 (Cvek stage 4 prognostic implications) |
| 9 | Therapeutic Intervention — types of intervention; administration (dosage, strength, duration); changes in intervention with explanations. | **Reported** | Manuscript, Report, First Visit–Second Visit, p.7–9 (TAP concentration/duration, PRF protocol, blood-clot-to-PRF change explained) |
| 10 | Follow-up and Outcomes — clinician- and patient-assessed outcomes; important follow-up test results; intervention adherence/tolerability; adverse and unanticipated events. | **Partially reported** | Manuscript, Follow-Up, p.9–10; Table 1 document, p.1 (referenced in Manuscript, p.10); Patient Perspective, p.15. No explicit statement of adverse/unanticipated events remains in the current manuscript (the previous reference to a gag-reflex event during radiography has been removed per reviewer request); Follow-Up, p.9–10, notes normal percussion/palpation and probing depths throughout, but this is not framed as an explicit adverse-event statement |
| 11 | Discussion — strengths and limitations of your approach; relevant medical literature; rationale for conclusions; one-paragraph take-away conclusion (without references). | **Reported** | Manuscript, Discussion, p.10–14 (incl. Limitations subsection, p.14); Conclusion, p.15 |
| 12 | Patient Perspective — the patient should share their perspective on the treatment(s) received. | **Reported** | Manuscript, Patient Perspective, p.15 |
| 13 | Informed Consent — the patient should give informed consent (provide if requested). | **Reported** | Manuscript, Report, p.7; Ethical Considerations, p.14–15; Consent for Publication, p.15–16 |
