# Supplementary material for: Regenerative Endodontic Therapy of an Immature Permanent Molar With Necrotic Pulp and Symptomatic Apical Periodontitis Using Platelet‐Rich Fibrin: A 52‐Month Follow‐Up Case Report
Source: Case Rep Dent. 2026 Aug 26;2026:2692780. doi: 10.1155/crid/2692780 (PMC13509411; doi:10.1155/crid/2692780)
Supplement: Supplementary file 1 [file CRID-2026-2692780-s001.zip › 2692780/PRICE_2020_Checklist_FINAL.docx]

**PRICE 2020 Checklist**

Manuscript: “Regenerative Endodontic Therapy of an Immature Permanent Molar with Necrotic Pulp and Symptomatic Apical Periodontitis Using Platelet-Rich Fibrin: A 52-Month Follow-Up Case Report.”

Journal: Case Reports in Dentistry

Nagendrababu V, Chong BS, McCabe P, Shah PK, Priya E, Jayaraman J, Pulikkotil SJ, Setzer FC, Sunde PT, Dummer PMH. PRICE 2020 guidelines for reporting case reports in Endodontics: a consensus-based development. Int Endod J. 2020;53(5):619–626.

| **Item** | **Description** | **Reported?** | **Location (document, page)** |
| --- | --- | --- | --- |
| 1a | The word 'case report(s)' must be included in the title. | **Reported** | Manuscript, Title page, p.1 |
| 1b | The area of interest (e.g., anatomy, disease, treatment) must be included briefly in the title. | **Reported** | Manuscript, Title page, p.1 |
| 2a | At least two relevant keywords, preferably MeSH terms, related to the content must be included. | **Reported** | Manuscript, Keywords, p.3 |
| 3a | The abstract must state how the report is novel and contributes to the literature and/or fills a gap in knowledge. | **Partially reported** | Manuscript, Abstract Background, p.2 (novelty/gap statement appears in the Introduction, p.4–6, but is not explicit within the Abstract itself) |
| 3b | The abstract must describe the main clinical findings, including symptoms and signs. | **Reported** | Manuscript, Abstract Case Report, p.2–3 |
| 3c | The abstract must describe the main radiographic/histological/laboratory/diagnostic findings. | **Reported** | Manuscript, Abstract Case Report, p.2–3 |
| 3d | The abstract must describe the main outcomes of treatment. | **Reported** | Manuscript, Abstract Case Report, p.2–3 |
| 3e | The abstract conclusion must contain the main take-away lesson(s). | **Reported** | Manuscript, Abstract Conclusion, p.2–3 |
| 4a | A background summary of the case with relevant information must be provided in the Introduction. | **Reported** | Manuscript, Introduction, p.4–6 |
| 5a | A clear statement that informed, valid consent was obtained from the patient(s) must be provided. | **Reported** | Manuscript, Report, p.7; Ethical Considerations, p.14–15; Consent for Publication, p.15–16 |
| 6a | The age of the patient(s) must be provided. | **Reported** | Manuscript, Report, p.6 (“7-year-old boy”) |
| 6b | The gender of the patient(s) must be provided. | **Reported** | Manuscript, Report, p.6 |
| 6c | The ethnicity of the patient(s), if relevant. | **Not reported** | Not mentioned in the manuscript. |
| 6d | The main concern/chief complaint of the patient(s). | **Reported** | Manuscript, Report, p.6 |
| 6e | The medical history of the patient(s), if relevant. | **Reported** | Manuscript, Report, p.6 (“medical history was non-contributory”) |
| 6f | The dental history of the patient(s), if relevant. | **Reported** | Manuscript, Report, p.6 (no previous dental treatment, regular OTC analgesic use) |
| 6g | The family history, if associated with the primary complaint. | **Not reported** | Not mentioned in the manuscript. |
| 6h | The psychosocial history, if associated with the primary complaint. | **Not reported** | Not mentioned at baseline (financial/logistical burden on the family is noted later, in Manuscript Limitations, p.14, but this concerns follow-up, not presenting psychosocial history) |
| 6i | Genetic information, relevant comorbidities and past interventions/outcomes, if relevant. | **Not reported** | Not applicable to this case (no known relevant genetic/comorbidity factors) stated in the manuscript |
| 6j | Extra-oral findings, if relevant. | **Not reported** | Not mentioned in the manuscript |
| 6k | General intra-oral findings (e.g., caries, restorations, periodontal condition, soft tissues). | **Reported** | Manuscript, Report, p.6 |
| 6l | Important dates/times (text, table, or figure) provided in chronological order. | **Reported** | Manuscript, Report/Follow-Up narrative, p.6–10; Table 1 document, p.1 (referenced in Manuscript text, p.10) |
| 6m | Diagnostic methods and results for the specific tooth/teeth. | **Reported** | Manuscript, Report, p.6 (cold test, EPT, periodontal probing, radiographs) |
| 6n | Diagnostic challenges, if any. | **Reported** | Manuscript, Report, p.6–7 (false-negative sensibility testing in immature teeth discussed) |
| 6o | Diagnostic reasoning, including other diagnoses considered. | **Partially reported** | Manuscript, Report, p.6–7 (possibility of partial residual pulp vitality is discussed; no other differential diagnoses are explicitly listed) |
| 6p | The active treatment(s)/intervention(s) performed. | **Reported** | Manuscript, Report, First Visit–Second Visit, p.7–9 |
| 6q | Modifications to the proposed treatment(s), if necessary. | **Reported** | Manuscript, Second Visit, p.7–8 (blood clot → PRF); Follow-Up, p.9 (delayed SSC placement) |
| 6r | Assessment method(s) for clinician- and patient-assessed outcomes and their results. | **Reported** | Manuscript, Follow-Up, p.9–10; Table 1 document, p.1 (referenced in Manuscript text, p.10); Patient Perspective, p.15 |
| 6s | Adverse and unanticipated events or consequences, if any. | **Partially reported** | No explicit statement of adverse or unanticipated events remains in the current manuscript. Follow-Up, p.9–10, notes the tooth remained asymptomatic with normal percussion/palpation and probing depths throughout 52 months, but this is not framed as an explicit adverse-event statement; a prior reference to a gag-reflex event during radiography has been removed from this version of the manuscript at reviewer request. |
| 7a | The specific treatment(s)/intervention(s) discussed with reference to the relevant literature. | **Reported** | Manuscript, Discussion, p.10–14 |
| 7b | The strengths of the report and its importance discussed with reference to the literature. | **Reported** | Manuscript, Discussion/Conclusion, p.10–15 (52-month follow-up in a literature where molar RET data beyond 24 months are scarce) |
| 7c | The limitations of the case report discussed. | **Reported** | Manuscript, Discussion – Limitations subsection, p.14 |
| 7d | The rationale for the conclusion(s) discussed. | **Reported** | Manuscript, Discussion, p.10–14; Conclusion, p.15 |
| 8a | Feedback from the patient on the treatment and care received, if relevant. | **Reported** | Manuscript, Patient Perspective, p.15 |
| 9a | Explicit conclusion(s)/take-away lesson(s) provided. | **Reported** | Manuscript, Conclusion, p.15 |
| 9b | Implications for clinical practice or future research provided. | **Reported** | Manuscript, Conclusion, p.15 (recommends prospective controlled studies) |
| 10a | Sources of funding/support and role of funders described. | **Reported** | Manuscript, Funding, p.16 |
| 11a | An explicit statement on conflicts of interest provided. | **Reported** | Manuscript, Conflict of Interest, p.16 |
| 12a | Equipment, software, and settings used to acquire the image(s) described. | **Partially reported** | Manuscript, Follow-Up, p.10 (ImageJ software with calibrated reference stated for radiographic measurement) |
| 12b | The reason the image was acquired and rationale for inclusion provided in text. | **Partially reported** | Implicit (images document each treatment stage, Manuscript, p.6–9) |
| 12c | The circumstances under which images were viewed/evaluated by the authors provided. | **Partially reported** | Manuscript, Follow-Up, p.10 (two examiners assessed radiographs using ImageJ with a calibrated reference) |
| 12d | Resolution and any magnification/modification of the image(s) described. | **Not reported** | Not stated in the Manuscript |
| 12e | Patient identifiers removed/anonymised. | **Partially reported** | Manuscript, Consent for Publication, p.15–16, states consent was obtained for publication of image. |
| 12f | Interpretation of findings from the image(s) provided in text. | **Reported** | Manuscript, Report, p.6–9; Discussion, p.10–14 |
| 12g | Legend describes the subject and specific feature(s) illustrated (plus age/gender/ethnicity if a person is shown). | **Partially reported** | Age/gender already established in Manuscript Report text, p.6 |
| 12h | Markers/labels used to identify key information, defined in legend or footnote. | **Partially reported** | In a separate Figure file uploaded with the submission, defined in legend. |
| 12i | Legend states whether pre-, intra-, or post-treatment, and how images over time were standardised. | **Partially reported** | Manuscript, Follow-Up, p.10, describes the standardisation method (paralleling technique/positioning jig) |
